# Abdominal Self-Stabbing: An Uncommon Type of Sharp Abdominal Trauma

**DOI:** 10.1155/2021/9917040

**Published:** 2021-07-21

**Authors:** Andrija Karačić, Borna Vojvodić

**Affiliations:** ^1^Department of General Surgery, University Hospital Sveti Duh, Sveti Duh, 64 Zagreb, Croatia; ^2^School of Medicine, University of Zagreb, Šalata 3, Zagreb, Croatia

## Abstract

Abdominal self-stabbing, a type of sharp abdominal trauma, is a rare form of attempted suicide. Such cases are not commonly seen in the emergency department, but a prompt and well-reasoned decision is essential in the management of these patients. We report a case of a SI-ASW and a literature review to show the management of the aforementioned condition.

## 1. Introduction

Self-inflicted stab wounds are an uncommon form of suicide, accounting for approximately 3% of all suicides [1], and the abdomen is the site of injury in approximately 30% of men and 13% of women in cases of self-stabbing [2]. Hence, self-inflicted abdominal stab wounds (SI-ASWs) are a rarely seen surgical emergency [3]. Stabbing as a form of sharp abdominal trauma can be associated with major abdominal injury, and it has been shown that self-inflicted stab wounds are usually as severe as stab wounds by assault [4]. Cases of SI-ASWs are mostly reported in medicolegal and psychiatric literature [5–7]; hence, there is inadequate published information relevant to the surgical management of SI-ASWs. We report a case of a SI-ASW and a literature review to elucidate the treatment options in the aforementioned condition. The following case report was prepared in accordance with the SCARE criteria [8]. Informed consent was obtained from the patient.

## 2. Case Presentation

**Figure 1:**
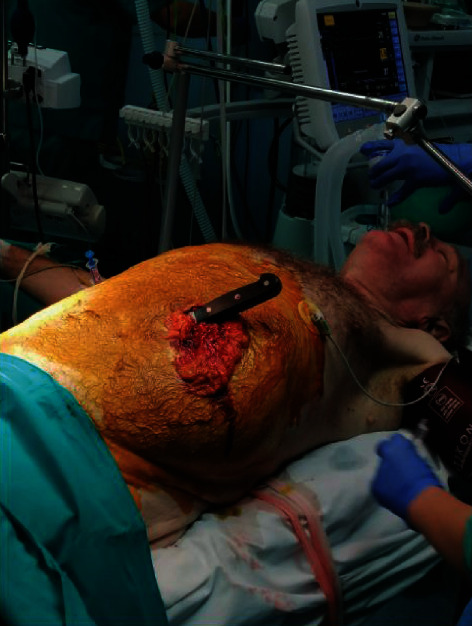
The patient on the operating table—the wound in the left hemiabdomen with the weapon in situ and omental evisceration are seen.

A 66-year-old male was brought into the emergency department of our hospital. He was found on the street unconscious with a kitchen knife stabbed into the left upper quadrant of the abdomen. After a short resuscitation, the patient was rushed to the operating room. In the left upper quadrant, a 15 cm long wound was seen with the knife inserted into the wound to the handle and omentum protruding through the wound (Figure 1). The wound was elongated distally to access the abdominal cavity. Approximately two liters of fresh and clotted blood was evacuated. The knife was found to be 15 cm long, and the tip of the knife was pointing to the left lateral abdominal wall. The blade penetrated the omentum, the gastrocolic ligament, and the transverse colon and the proximal one-third of the transverse mesocolon. All bleeding sites were ligated. During exploration, an additional mesenteric lesion was found, ligated, and resected. No other abdominal organs or major blood vessels were injured. Omentectomy, appendectomy, and resection of a part of the ascending and transverse colon with a protective cecostomy were performed. The patient's postoperative recovery was uneventful. Upon awakening, the patient confessed that the reason for the stabbing was attempted suicide. The patient was subsequently referred to a psychiatric clinic. After 6 months, colon continuity was established through ileocolic anastomosis. Follow-up was routine.

## 3. Discussion

We encountered a very rare case of a self-inflicted abdominal stab wound (SI-ASW) with the weapon in situ. Immediate and successful management led to a fast and uneventful recovery.

Considering the epidemiology of SI-ASWs, our case is a typical patient with this condition. SI-ASWs are most common among men, with percentages in the literature of approximately 70% [9]. The reported median age of patients with SI-ASWs is 40 years [3]. Common risk factors associated with SI-ASWs are psychiatric disorders, especially alcohol and drug abuse [5]. Our patient did not report any alcohol or drug abuse. Attempted suicide was the result of an underlying psychiatric disorder requiring treatment in a specialized institution after successful postoperative recovery.

Kitchen knives, as in our case, are the most frequently used weapon for SI-ASWs, probably due to their broad availability [10].

Most patients with SI-ASWs stab themselves multiple times with an average number of 1.5 stabs [3].

The location of the stabbing is most frequently in the right hemiabdomen, which is assumed to be since most people are right handed [3]. However, there are geographical differences noted. In a Japanese study, the most prominent site was the periumbilical and epigastric region, probably a result of the Japanese “hara-kiri” tradition of a transverse cut when attempting suicide [11].

The most commonly injured organs are the liver, small bowel, and colon [12]. Extra-abdominal areas are also frequently wounded, including areas such as the wrists, arms, legs, and neck. These are often hesitation wounds [13].

Upon arrival of a patient with a SI-ASW at the emergency department, it is essential to immediately evaluate the grade of injury and shape a management strategy accordingly. First, an advanced trauma life support (ATLS) primary survey and thorough physical examination are necessary [14] to classify the severity of the SI-ASW.

In the case of minor injuries, diagnostic work-up can be commenced. Local wound exploration can be performed to determine peritoneal perforation. A further option is diagnostic peritoneal lavage (DPL), which is a simple and inexpensive but invasive and somewhat outdated method [14]. Different radiological imaging methods, such as ultrasound or computed tomography, can be used to assess the type, localization, and depth of the injury. If the patient is hemodynamically stable and peritoneal penetration or hollow viscus perforation is suspected, diagnostic laparoscopy can be performed [15]. Although laparoscopy was avoided in abdominal trauma for a long time [16], the trend has moved away from mandatory exploratory laparotomy to diagnostic and therapeutic laparoscopy for anterior abdominal wounds [17]. Diagnostic laparoscopy can offer definitive information on the extent of injury and, in certain circumstances, can also be therapeutic [18]. In addition to the well-known benefits of laparoscopy, such as faster recovery and lower morbidity and mortality [14], it can significantly reduce the rate of unnecessary laparotomies [19]. Up to 40% of diagnostic laparotomies are associated with substantial postoperative complications [20].

Emergency laparoscopy requires the availability of proper technological equipment [21] and a surgeon who is well trained and experienced in laparoscopy [22]. Other limitations of laparoscopy for SI-ASWs are different technical difficulties, such as large blood collections [23]. Patient-related contraindications for laparoscopy are serious cardiopulmonary dysfunction, septic shock, severe hypovolemic shock, traumatic brain injury, inability to tolerate pneumoperitoneum, diffuse peritonitis, severe COPD, obvious evisceration, impalement, intra-abdominal adhesions, and history of laparotomy [23].

In the case of major injury with demonstrated peritoneal violation, the patient should undergo exploratory laparotomy, as laparoscopy has not been shown to be superior to laparotomy in such cases [24]. If a SI-ASW is associated with hemodynamic instability, the patient should be rushed to exploratory laparotomy immediately. Hemodynamic instability is the major contraindication for laparoscopy in abdominal trauma [23].

It is important to note that if the weapon is in situ, as in our case, extraction should be performed only in the operating room where the wound can be explored and immediate hemostasis can be achieved.

The literature shows that while SI-ASWs can be associated with major injuries, they are rarely lethal [25]. The reported mortality of this injury is approximately 3% (references here). As with our patient, most patients recover from this type of trauma if they arrive alive at the emergency department. They rarely succumb to this type of self-inflicted injury.

In conclusion, we report an uncommon form of sharp abdominal trauma that was well managed and had a good outcome. If a patient with a SI-ASW arrives at an emergency department quickly and receives prompt and appropriate management, he/she will most likely survive without major consequences.

## Data Availability

No data was used to support this study.
